# Case Report: A rare case of esophageal squamous cell carcinoma with metastases to the pancreas and stomach

**DOI:** 10.3389/fonc.2026.1837759

**Published:** 2026-06-11

**Authors:** Yuhang Lv, Yanting Jiang, Kaiyuan Hui, Xiaodong Jiang

**Affiliations:** 1 Department of Oncology, Lianyungang Clinical College of Nanjing Medical University, Lianyungang, China; 2 Department of Oncology, The Affiliated Lianyungang Hospital of Xuzhou Medical University, Lianyungang, China

**Keywords:** case report, esophageal squamous cell carcinoma, pancreatic metastasis, precision therapy, tumor dissemination

## Abstract

Esophageal cancer is a highly aggressive malignancy associated with a generally poor clinical prognosis. While distant metastases are common, the metastasis of esophageal squamous cell carcinoma (ESCC) to the pancreas and stomach is extremely rare. We report the case of a 62-year-old male with a history of ESCC who presented with an incidentally discovered pancreatic mass on imaging 37 months after the completion of radiotherapy. The patient subsequently underwent radical surgical resection. Postoperative histopathological and immunohistochemical analyses confirmed the presence of pancreatic and gastric metastases originating from the primary ESCC. For subsequent disease management, the patient received a systemic regimen comprising immune checkpoint inhibitors combined with platinum-based chemotherapy. This case underscores the importance of considering rare metastases in the differential diagnosis of new pancreatic lesions in patients with a history of ESCC, even following a prolonged disease-free interval. Furthermore, it suggests that aggressive surgical resection combined with postoperative immunochemotherapy may represent a viable multidisciplinary therapeutic strategy for managing such atypical metastatic presentations.

## Introduction

Esophageal cancer is one of the most aggressive malignancies worldwide, ranking as the 11th most common incidence cancer and the seventh leading cause of cancer-related mortality globally (1). The predominant histological subtype, esophageal squamous cell carcinoma (ESCC), is characterized by a high propensity for local invasion and early hematogenous and lymphatic dissemination, conferring a generally poor overall prognosis.

Clinically, the most frequent sites of distant metastasis for ESCC include the lymph nodes, lungs, liver, bones, adrenal glands, and brain (2). Conversely, metastasis of ESCC to the pancreas is an extremely rare clinical entity. Because the vast majority of pancreatic malignancies are primary tumors, pancreatic metastases are highly uncommon, accounting for less than 2% of all pancreatic neoplasms (3). Among these already rare secondary pancreatic lesions, those originating from esophageal cancer are exceptionally scarce.

In light of this rarity, we present a unique case of a patient with ESCC who developed isolated pancreatic and gastric metastases after achieving a prolonged disease-free interval (DFI) of 37 months following curative-intent radiotherapy. The patient successfully underwent an *en bloc* multi-organ resection, which included a distal pancreatectomy, followed by postoperative adjuvant systemic therapy with a guideline-recommended immunochemotherapy regimen (an immune checkpoint inhibitor combined with platinum-based doublet chemotherapy). By reporting this case and reviewing the relevant literature, we aim to heighten clinical awareness of pancreatic metastases originating from ESCC, highlight key considerations for differential diagnosis, and explore optimal multidisciplinary treatment (MDT) strategies for this atypical metastatic presentation.

## Case presentation

### Initial diagnosis and treatment of the primary lesion

A 62-year-old male presented in September 2022 with dysphagia and retrosternal pain. The patient had a 30-year history of heavy smoking (20 cigarettes per day) and chronic alcohol consumption. At the time of initial presentation, his known comorbidities included essential hypertension, which had been well-controlled for 10 years; notably, he had no prior diagnosis of diabetes mellitus, chronic liver or kidney diseases, or other chronic comorbidities. The patient denied any history of hereditary cancer syndromes, germline genetic alterations, or exposure to carcinogenic occupational factors. Family history was unremarkable, with no first- or second-degree relatives diagnosed with gastrointestinal malignancies or other hereditary tumors. Psychosocially, the patient had a stable marital status, normal social functioning, and no history of mental illness or adverse psychosocial stressors.

Upper endoscopy revealed an irregular, ulcerative lesion located 28–34 cm from the incisors (**Figures 1a, b**). Histopathological examination of the biopsy specimen confirmed a diagnosis of ESCC (**Figure 1c**). Subsequent computed tomography (CT) and positron emission tomography-CT (PET-CT) scans demonstrated mural thickening of the middle and lower esophageal segments (**Figure 1d**) with significant glucose hypermetabolism (SUVmax 7.2). No evidence of local lymph node involvement or distant organ metastasis was detected. The clinical staging was thus determined to be localized disease.

**Figure 1:**
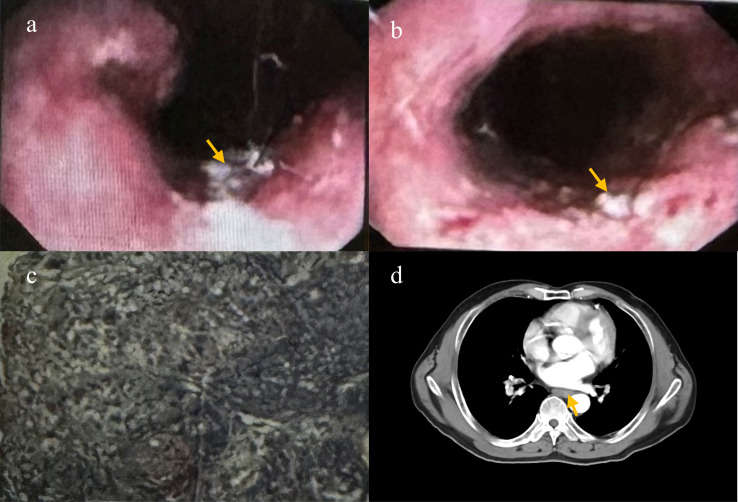
**(a, b)** Upper endoscopy revealing an irregular ulcerative lesion 28–34 cm from the incisors (indicated by the arrow). **(c)** Histological analysis of the biopsy specimen with hematoxylin and eosin staining, confirming esophageal squamous cell carcinoma (magnification × 100). **(d)** CT scan demonstrating mural thickening of the middle and lower esophagus (indicated by the arrow).

Shortly after the ESCC diagnosis, the patient’s clinical management was complicated by the emergence of symptomatic cardiovascular disease. In October 2022, he was diagnosed with severe coronary artery disease (CAD), featuring approximately 80% stenosis of the left anterior descending artery, which necessitated coronary stent implantation that same month. Following this intervention, a multidisciplinary team (MDT) consultation determined that the risks associated with radical esophagectomy or aggressive concurrent chemoradiotherapy were prohibitive due to his recent cardiac event and required antiplatelet therapy. Consequently, the patient underwent definitive curative-intent intensity-modulated radiation therapy (IMRT) with a total dose of 60 Gy delivered in 30 fractions in November 2022. The gross tumor volume (GTV) was delineated as the primary esophageal tumor with a length of 7.5 cm. The clinical target volume (CTV) was contoured with a radial margin of 0.8 cm, a cranial longitudinal margin of 2.5 cm, and a caudal longitudinal margin of 2.0 cm, while the planning target volume (PTV) was set as the CTV with an additional 0.5 cm isotropic margin. Notably, the celiac axis and left gastric artery regions were not included in the irradiation field. Following this treatment, the patient’s dysphagia significantly improved, and the primary tumor was considered well-controlled.

### Disease relapse and detection of pancreatic lesion

In January 2026, approximately 37 months following the completion of his initial radiotherapy, the patient presented with spontaneous, persistent dull pain in the left upper abdominal quadrant radiating to the back. The patient had poor follow-up compliance and did not receive regular imaging surveillance after radiotherapy due to complete relief of esophageal symptoms. An initial non-contrast abdominal CT scan revealed a mass involving the body and tail of the pancreas (**Figure 2a**). For further characterization and systemic evaluation, a whole-body PET-CT scan was performed. This imaging demonstrated a low-density mass in the pancreatic body exhibiting focal fluorodeoxyglucose (FDG) hypermetabolism (SUVmax 6.9) (**Figure 3**). The pancreatic lesion was anatomically contiguous with the gastric lesion without isolated FDG uptake. Based on these clinical and radiographic findings, the lesion was initially presumed to be a primary pancreatic carcinoma. Additionally, multiple mildly enlarged retroperitoneal lymph nodes were observed; however, these lacked significant metabolic activity, suggestive of benign reactive changes. No other hypermetabolic lesions indicative of distant metastasis were detected. Notably, there was no radiologic evidence of local recurrence at the primary esophageal site, confirming a sustained complete response to the prior radiotherapy.

**Figure 2:**
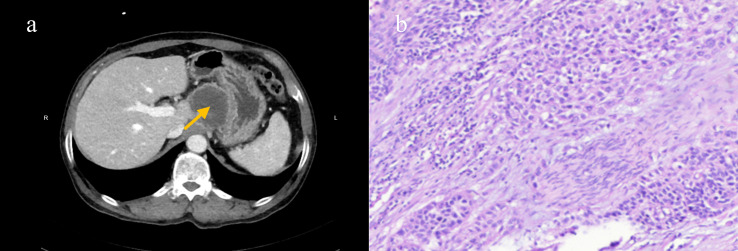
**(a)** CT scan showing a mass in the body and tail of the pancreas (indicated by the arrow). **(b)** Representative image from histopathological examination of biopsy specimens stained with hematoxylin and eosin, confirming the diagnosis of pancreatic squamous cell carcinoma (magnification × 100).

**Figure 3:**
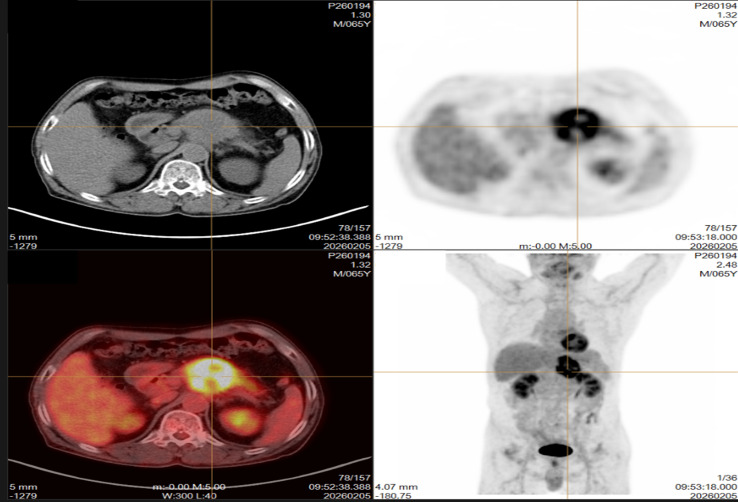
PET-CT result showing a slightly hypodense mass in the body of the pancreas with increased glucose metabolism.

### Surgical intervention and pathological diagnosis

Due to the localized nature of the lesion, the patient underwent a combined organ resection in February 2026 to establish a definitive pathological diagnosis and alleviate obstruction and pain. The surgical procedure included resection of the pancreatic body and tail, splenectomy, proximal gastrectomy with esophagogastric anastomosis, and abdominal lymph node dissection. Postoperative histopathological examination revealed infiltration of poorly differentiated cancer cells with extensive necrosis in the pancreatic parenchyma, accompanied by nerve invasion (**Figure 2b**). Focal malignant tissue was also identified in the subserosal layer of the stomach. Immunohistochemical (IHC) staining confirmed the squamous epithelial origin of the tumor cells, showing strong positivity for CK5/6 (3+), P40 (3+), and CK19 (3+), while adenocarcinoma and intestinal markers CK7 and CK20 were negative. The Ki-67 proliferation index was 70%. No tumor involvement was detected at the surgical margins, and all 12 peripancreatic lymph nodes were free of metastases (0/12). Considering the patient’s history of esophageal cancer and the IHC profile, the final diagnosis was ESCC with metastases to the pancreas and stomach.

### Postoperative management and follow-up

The patient recovered well postoperatively. Given the aggressive clinical behavior and high metastatic potential of this disease, first-line systemic therapy was planned to control micrometastatic disease and delay progression. Following the 2025 Chinese Society of Clinical Oncology (CSCO) Guidelines for the Diagnosis and Treatment of Esophageal Cancer, a comprehensive treatment regimen was formulated combining an immune checkpoint inhibitor with platinum-based dual-agent chemotherapy. The regimen consists of sintilimab 200 mg intravenously on Day 1, cisplatin 75 mg/m² intravenously on Day 1, and paclitaxel 175 mg/m² intravenously on Day 1, with each cycle lasting 21 days.

At the last follow-up in April 2026, the patient had completed 2 cycles of the combination regimen, with an Eastern Cooperative Oncology Group (ECOG) performance status of 1. No severe treatment-related adverse events (grade ≥3) were observed. Imaging re-examination showed no evidence of tumor recurrence or new distant metastasis, and the patient achieved stable disease (SD) with good clinical tolerance. The patient remains under close clinical monitoring to dynamically evaluate long-term efficacy and monitor potential adverse effects. The timeline of the patient’s onset, diagnosis, and treatment is summarized in **Figure 4**.

**Figure 4:**
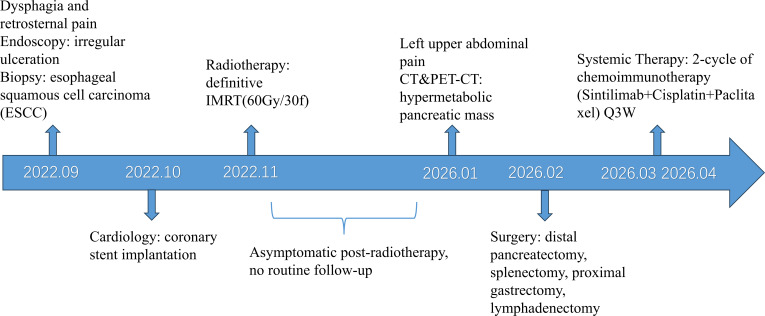
Timeline of the patient’s clinical course.

## Discussion

Metastasis of ESCC to the pancreas is an exceptionally rare clinical event. To date, only a limited number of isolated cases have been documented in the literature. This report describes a case of a 62-year-old man who developed metachronous metastases to both the pancreas and stomach following a prolonged DFI after definitive radiotherapy for primary ESCC. To further elucidate the clinical course and characteristics of this uncommon presentation, we conducted a literature review of articles indexed in the PubMed database from 2011 to 2025, using the following keywords: esophageal squamous cell carcinoma, pancreatic metastasis, and secondary tumors. Our search identified 14 previously reported cases of ESCC with pancreatic metastasis, which are summarized in **Table 1**.

**Table 1:** Reported cases of pancreatic metastasis from ESCC.

Year of publication	Age (years)	Sex	Synchronous/metachronous	Treatment	Recurrence	Ref
2011	59	F	Metachronous	DP+5-FU	No	(4)
2013	73	M	Synchronous	Supportive care	Not mentioned	(5)
2013	58	M	Synchronous	DP+FP	No	(6)
2014	68	M	Metachronous	DP+FP	No	(7)
2014	60	M	Synchronous	TC	Not mentioned	(8)
2018	67	M	Synchronous	Not specified	Not mentioned	(9)
2019	81	F	Metachronous	DP	No	(10)
2019	67	M	Synchronous	Palliative care	Not mentioned	(11)
2021	67	M	Synchronous	Palliative care	Not mentioned	(12)
2023	69	M	Synchronous	mFOLFOX6+Nivo+PTX	No	(13)
2024	75	M	Metachronous	DP	No	(14)
2024	53	F	Metachronous	Cis+Cap+5-FU+RT	Under continuous observation	(15)
2024	66	M	Synchronous	FOLFOX+ Nivo	No	(16)
Present case	62	M	Metachronous	DP+Sintilimab+PTX+Cis	Under continuous observation	

F, female; M, male; DP, distal pancreatectomy; 5-FU, 5-fluorouracil; FP, 5-fluorouracil plus cisplatin; TC, taxotere plus cyclophosphamide; mFOLFOX, modified folinic acid/fluorouracil/oxaliplatin; Nivo, nivolumab; PTX, paclitaxel; Cis, cisplatin; Cap, capecitabine; RT, radiotherapy.

The precise mechanisms underlying pancreatic metastasis from ESCC remain a subject of ongoing debate. While metastasis is traditionally attributed to local lymphatic spread, venous pathways, or systemic hematogenous dissemination, anatomical and hemodynamic principles alone do not fully account for this atypical phenomenon. Given that the pancreas weighs only 120–180 grams and receives a relatively small proportion of cardiac output, the probability of circulating tumor cells (CTCs) becoming mechanically trapped within the pancreatic parenchyma is inherently low.

Consequently, the marked organotropism observed in these rare cases is better elucidated by the “seed and soil” hypothesis, originally proposed by Paget and recently expanded by Sellner et al. to explain isolated pancreatic metastases (17, 18). According to this paradigm, systemic dissemination is merely the initial step; establishing a clinically detectable secondary tumor requires high biological compatibility between the CTCs (“seeds”) and the specific microenvironment of the host organ (“soil”). If the characteristics of the embolized cancer cells do not align with the target organ, they are typically eliminated by local immune surveillance or enter a state of dormancy. Thus, only specific ESCC subclones that have undergone microevolutionary adaptations can establish a pre-metastatic niche, explaining both the rarity of such metastases and the often-prolonged DFI observed.

Given that the management of primary and secondary pancreatic malignancies differs fundamentally, establishing definitive histopathological evidence is paramount. Recent literature strongly advocates for endoscopic ultrasound-guided fine-needle aspiration (EUS-FNA) to obtain preoperative tissue samples, potentially sparing patients from unnecessary and highly invasive pancreatic resections (19). In the present case, however, the patient underwent immediate multivisceral resection. This clinical decision was driven by a high index of suspicion for a primary pancreatic malignancy involving the stomach, necessitating urgent intervention for both diagnostic confirmation and local tumor control. Postoperative analysis revealed high expression of squamous markers (CK5/6+, P40+, and CK19+) and a complete absence of glandular markers (CK7- and CK20-). These findings definitively refuted the initial suspicion of primary pancreatic ductal adenocarcinoma (PDAC), underscoring the indispensable role of immunohistochemistry (IHC) in distinguishing pancreatic metastases from primary malignancies.

There is currently no established consensus on the treatment strategy for ESCC with pancreatic metastasis. Based on our comprehensive review of 14 cases, prognosis appears to depend largely on the extent of systemic involvement and the timing of metastatic presentation. For patients with synchronous or widespread disease, systemic palliative therapy remains the cornerstone of treatment. Conversely, for patients with highly localized, isolated subchronic metastases and a longer DFI—such as our patient—aggressive surgical intervention (e.g., distal pancreatectomy) has been shown to provide a tangible survival benefit. Achieving R0 resection not only effectively reduces the tumor burden but also mitigates the risk of debilitating complications, such as biliary or gastrointestinal obstruction.

Beyond local surgical control, the systemic nature of metastatic ESCC mandates robust medical management. With the clinical integration of immune checkpoint inhibitors (ICIs), the paradigm for systemic therapy has shifted. While historical protocols relied exclusively on platinum-based chemotherapy, emerging data suggest that the addition of PD-1 inhibitors can synergistically enhance antitumor efficacy (20). In alignment with the 2025 CSCO guidelines (21), our patient is currently receiving a multimodal postoperative regimen of sintilimab, cisplatin, and paclitaxel. This comprehensive approach aims to eradicate occult micrometastases and extend the interval to disease recurrence.

In summary, although pancreatic metastasis from ESCC represents a specific clinical finding, it must be included in the differential diagnosis of newly discovered pancreatic masses, particularly in patients with a prior history of ESCC, even after a prolonged disease-free interval. A tailored multidisciplinary approach, combining meticulous pathological verification, selective curative surgery, and modern immunotherapy, offers the most promising pathway to improved outcomes for this extremely rare patient population.

## Data Availability

The original contributions presented in the study are included in the article/supplementary material. Further inquiries can be directed to the corresponding authors.
